# Decrements of body mass index are associated with poor outcomes of idiopathic pulmonary fibrosis patients

**DOI:** 10.1371/journal.pone.0221905

**Published:** 2019-10-04

**Authors:** Tejaswini Kulkarni, Kaiyu Yuan, Thi K. Tran-Nguyen, Young-il Kim, Joao A. de Andrade, Tracy Luckhardt, Vincent G. Valentine, Daniel J. Kass, Steven R. Duncan

**Affiliations:** 1 Department of Medicine, Division of Pulmonary, Allergy, and Critical Care Medicine, University of Alabama at Birmingham, Birmingham, Alabama, United States of America; 2 Department of Medicine, Division of Preventive Medicine, University of Alabama at Birmingham, Birmingham, Alabama, United States of America; 3 Birmingham VA Medical Center, Birmingham, Alabama, United States of America; 4 Department of Medicine, Division of Pulmonary, Allergy, and Critical Care Medicine, University of Pittsburgh, Pittsburgh, Pennsylvania, United States of America; Vanderbilt University Medical Center, UNITED STATES

## Abstract

**Background:**

The processes that result in progression of idiopathic pulmonary fibrosis (IPF) remain enigmatic. Moreover, the course of this disease can be highly variable and difficult to accurately predict. We hypothesized analyses of body mass index (BMI), a simple, routine clinical measure, may also have prognostic value in these patients, and might provide mechanistic insights. We investigated the associations of BMI changes with outcome, plasma adipokines, and adaptive immune activation among IPF patients.

**Methods:**

Data were analyzed in an IPF discovery cohort (n = 131) from the University of Pittsburgh, and findings confirmed in patients from the University of Alabama at Birmingham (n = 148). Plasma adipokines were measured by ELISA and T-cell phenotypes determined by flow cytometry.

**Results:**

Transplant-free one-year survivals in subjects with the greatest rates of BMI decrements, as percentages of initial BMI (>0.68%/month), were worse than among those with more stable BMI in both discovery (HR = 1.8, 95%CI = 1.1–3.2, p = 0.038) and replication cohorts (HR = 2.5, 95%CI = 1.2–5.2, p = 0.02), when adjusted for age, baseline BMI, and pulmonary function. BMI decrements >0.68%/month were also associated with greater mortality after later lung transplantations (HR = 4.6, 95%CI = 1.7–12.5, p = 0.003). Circulating leptin and adiponectin levels correlated with BMI, but neither adipokine was prognostic *per se*. BMI decrements were significantly associated with increased proportions of circulating end-differentiated (CD28^null^) CD4 T-cells (CD28%), a validated marker of repetitive T-cell activation and IPF prognoses.

**Conclusions:**

IPF patients with greatest BMI decrements had worse outcomes, and this effect persisted after lung transplantation. Weight loss in these patients is a harbinger of poor prognoses, and may reflect an underlying systemic process, such as adaptive immune activation.

## Introduction

The median survival of patients with idiopathic pulmonary fibrosis (IPF) is three-to-four years, but the courses of individuals with this lung disease are highly variable [1]. An accurate means to predict IPF patient outcomes could enable health care providers to better optimize the timing of lung transplantation referrals, encourage compliance with medications that have bothersome side effects among those at greatest risk, and identify particular subpopulations for experimental clinical trials or timely end-of-life counseling. Accordingly, the development of prognostic indicators for IPF is an active area of interest [2–6]. Nonetheless, currently available prognostic instruments do not have perfect accuracy or universal applicability [2, 3, 7–10].

Body mass index (BMI) is routinely assessed in clinic practice, and is available in any facility that can measure patient heights and weights. Associations between BMI and prognoses have been reported among patients with chronic obstructive pulmonary disease (COPD) [11, 12], and several other disorders such as rheumatoid arthritis [13] and heart failure [13, 14]. While recent studies have also linked BMI to survival in IPF patients [15–18], the mechanism(s) associated with weight loss in this population have not been explored. Several factors have been causally implicated in pathological BMI decrements among other disease populations such as chronic inflammation, oxidative stress, and adipose tissue metabolism [19–21].

We have noted several anecdotal examples in our clinical practices wherein unexplained weight loss in patients with IPF seemed to portend poor prognoses. Consequently, we conducted analyses here to systematically test the hypothesis that dynamic changes of BMI may be associated with decreased survival among IPF patients. We postulated too that BMI reductions in this population might be related to an underlying systemic process(es) and not merely a simple, direct correlate of pulmonary physiology *per se*.

## Materials and methods

### Subjects

Clinical information about patients with ≥2 BMI measures between May 2000 and June 2014 was abstracted from prospectively recorded research registries at the University of Pittsburgh Medical Center (UPMC) and the University of Alabama at Birmingham (UAB). All subjects fulfilled IPF consensus criteria [1]. The Institutional Review Boards for Human Research approved this project, including waiver of informed consent at both institutions (UAB: E150318008 and UPMC: MOD0610029-14).

### Clinical variables

Measurements of forced vital capacity (FVC) and diffusion capacity for carbon monoxide (D_L_CO), expressed as percentages of predicted values (%FVC and %D_L_CO, respectively), were performed per guidelines [22].

BMI (kg/m^2^) was calculated as weight/height^2^. BMI measurements were made using medical-grade scales and were performed as routine clinical practices in dedicated interstitial lung disease clinics by trained personnel. BMIs at the subject's last clinic visit prior to their death (all-cause mortality), lung transplantation, or June 1, 2014, were denoted as BMI_last_. Measurements made at clinic visits closest to the one-year prior to BMI_last_ were designated as BMI_first_. Percentage changes of BMI during these intervals (%BMI_delta_) were calculated as ([BMIlast−BMI_first_]/BMI_first_) x 100. Rates of relative changes were calculated by dividing %BMI_delta_ by the interval (in months) during which these changes occurred (i.e., %BMI_delta_/month).

The primary endpoint of the study was one-year transplant-free survival (TFS), defined as the interval (for up to 12 months) after BMI_last_ that subjects were alive and had not undergone lung transplantation. Deaths from any cause or transplantations were considered uncensored events. Transplant-free survivors were censored at one-year after BMI_last_, as were any cases in this subpopulation that were lost to follow-up during the 12-month observation. Mortality ascertainments were made by review of clinic and hospital records, searches of internet databases (including obituary listings), and telephone interviews.

To perform survival analyses, we dichotomized discovery cohort subjects into the quartile with the greatest rates of weight loss *vs*. the remaining 75% who had lesser BMI reductions. The cut off value distinguishing these two categories was 0.68%/month in the discovery cohort and this value was applied to similarly dichotomize subjects in the validation cohort, and those who underwent lung transplantation.

### Circulating adipokines

Leptin and adiponectin were measured in plasma specimens by ELISA (R&D Systems, Minneapolis, MN) according to the manufacturer's instructions.

### Flow cytometry

Peripheral blood mononuclear cells (PBMNC) were isolated from phlebotomy specimens by density gradient centrifugation [23]. CD4 T-cell CD28 expression was determined by methods detailed previously [23, 24]. CD28% values were defined as the proportion of circulating CD4 T-cells that co-express CD28 [23, 24]. Downregulation of CD28 on surfaces of circulating CD4 T-cells is a specific and validated marker of repeated antigen encounters and adaptive immune activation (23,24).

### Statistical analysis

Descriptive statistics were presented as means ± SD and frequencies, or as proportions for categorical variables. Continuous variables were compared by Mann-Whitney. Bivariate associations were evaluated by Chi-square or Fisher’s exact tests. Intergroup differences in time-to-event were evaluated by Kaplan-Meier survival analyses and log-rank tests. Multivariable cox proportional-hazards regression analyses were used to assess TFS after accounting for age and baseline BMI as a continuous variable and absolute decline by 10 percentage points of %FVC and %D_L_CO, over the same interval as the measured BMI decrement, as categorical variables. These physiological variables have often been associated with survival in IPF [25] and hence, were included in the multivariable models evaluating associations between TFS and decrements of %BMI_delta_ and CD28 expression. Associations between continuous variables (BMI and adiponectin levels) were established by Spearman correlations. P-values <0.05 were considered significant. Statistical analyses were performed using SAS software^TM^ (SAS 9.4).

## Results

### Subject characteristics

Characteristics of the subjects are detailed in Table 1.

**Table 1:** Demographics and baseline characteristics of subjects.

	UPMC Discovery Cohort (n = 131)	UAB Validation Cohort (n = 148)	p-value
**Age (mean, years)**	68.9 ± 8.9	65.3 ± 8.9	<0.01
**Gender (male)**	101 (77%)	100 (68%)	0.06
**Race (white)**	129 (99%)	138 (94%)	0.01
**Current/Former smoker**	96 (74%)	105 (72%)	0.07
**BMI**_**first**_ **(m**^**2**^**)**	29.8 ± 5.0	29.9 ± 6.1	0.90
**BMI Interval (months)**	10.6 ± 3.8	10.7 ± 4.4	0.50
**%Predicted FVC (baseline)**	64 ± 17	59 ± 18	0.03
**%Predicted D**_**L**_**CO (baseline)**	48 ± 17	44 ± 14	0.06

Data represent absolute numbers, and/or means ± SD, and/or percentages. UPMC: University of Pittsburgh, UAB: University of Alabama at Birmingham, BMI: Body Mass Index; BMI Interval denotes elapsed time between BMI measures (BMI_first_ and BMI_last_, respectively); FVC: Forced Vital Capacity; D_L_CO: Diffusing Capacity for Carbon Monoxide; Current/former smoker denotes subjects with ≥ 5 pack-years of cigarette smoking.

### Discovery cohort

**Fig 1:**
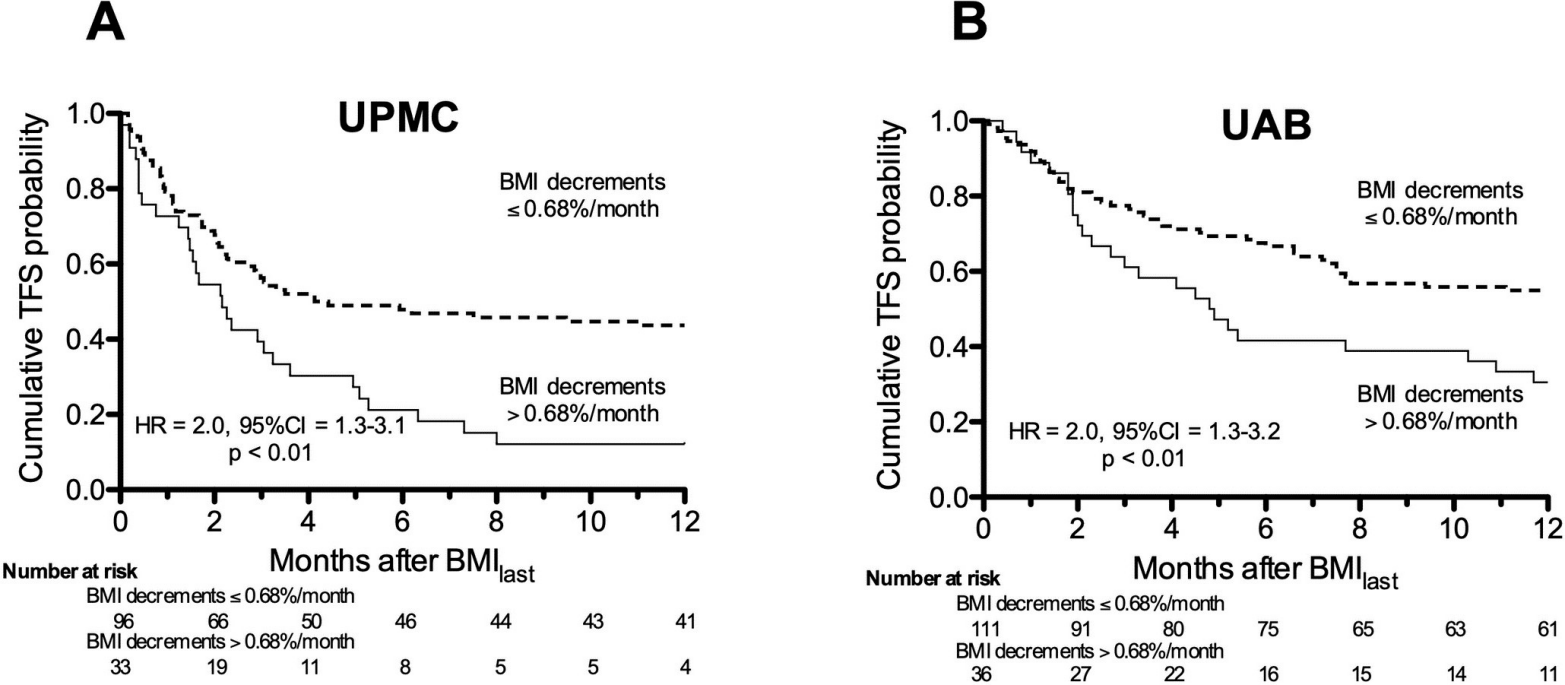
Transplant-free survival dichotomized by BMI decrement. **A)** Transplant-free survival (TFS) among the University of Pittsburgh Medical Center (UPMC) discovery cohort IPF patients was worse in the quartile with the greatest decrements of BMI rates (%BMI_delta_/month). The cut-off value that defined this worst performing quartile was >0.68%/month. **B)** TFS among the University of Alabama at Birmingham (UAB) replication cohort was similarly reduced among the IPF patients with BMI decrements >0.68%/month.

Among the discovery cohort (n = 131), 38 subjects (29%) underwent transplantation, and there were 47 (36%) pre-transplant deaths. One subject was lost to follow-up 7.5 months after BMI_last_ and was censored at that time, whereas all other participants were observed for 12 months, or until their deaths or transplantations. %BMI_delta_, adjusted for the interval over which the weight loss occurred (%BMI_delta_/month) was -0.42 ± 0.70%/month for those with transplants or deaths *vs*. -0.09 ± 0.44%/month among non-transplanted survivors (p<0.01). TFS was significantly worse among subjects in the quartile with the greatest rates of BMI decrements (which corresponded to BMI reductions >0.68%/month) compared to patients with a more stable BMI (Fig 1A). The increased risk with greater BMI decrements also persisted in the multivariate regression analysis adjusting for age, pulmonary function and initial BMI (HR = 1.8, 95%CI = 1.1–3.2, p = 0.038).

In order to exclude the possibility these results might be confounded by the intentional weight loss of a few patients, in order for them to become listed for transplantation, we conducted a *post hoc* analyses that omitted patients with initial BMI >30 (this is generally the upper limit BMI for lung transplantation eligibility). Despite now having a smaller study cohort (n = 93), the TFS among them who had the greatest BMI_delta_ decrements (>0.68%/month) was still significantly worse than those with more stable BMI (HR = 2.1, 95%CI = 1.1–3.8, p = 0.02).

To further exclude the possibility of bias due to intentional weight loss, which would have been a necessary prelude to their transplantations, we also performed an analysis that excluded the patients who had lung transplantation at any time. Nonetheless, survival in the subjects with the greatest rates of BMI_delta_ decrements remained significantly worse than those with more stable BMI (HR = 2.9, 95%CI = 1.6–5.2, p = 0.0002).

Yet another *post hoc* analyses, in which lung transplantations were instead treated as censored events, again showed increased risk of death among those IPF patients with the greatest BMI_delta_ decrements (HR = 2.8, 95%CI = 1.5–5.0, p = 0.003).

### Validation cohort

The validation cohort was fortuitously well-matched in most respects to the initial discovery subjects (Table 1). Most of the differences between these two groups were comparatively small and perhaps of greater mathematical significance than biological importance.

Eighty-nine (89) of the 148 validation cohort subjects (60%) subjects died prior to transplantation and 23 others (16%) had lung transplantations. All subjects in this cohort were observed for 12 months or until death or transplantation (i.e., there were no censored events). Time-adjusted rates of %BMI_delta_ were -0.41 ± 0.84%/month *vs*. -0.06 ± 0.74%/month among subjects with and without adverse events, respectively (p<0.01). Stratification of the replication cohort into subjects with BMI_delta_ reductions greater than or less than the 0.68%/month cut off value found in the discovery cohort again showed subjects with more extreme weight loss had a 2-fold increased risk of a worse outcomes (Fig 1B). Multivariable adjustments for age, pulmonary function, and initial BMI did not alter the association between decrements of %BMI_delta_ and TFS at one year in this cohort (HR = 2.5, 95%CI = 1.2–5.2, p = 0.02).

### Association of BMI with Post-transplant survival

We also examined effects of BMI changes on mortality after subsequent lung transplantations in the UPMC discovery subjects. The proportion of subjects who underwent lung transplantation among the UAB cohort was too small to permit meaningful survival analyses in this group.

**Fig 2:**
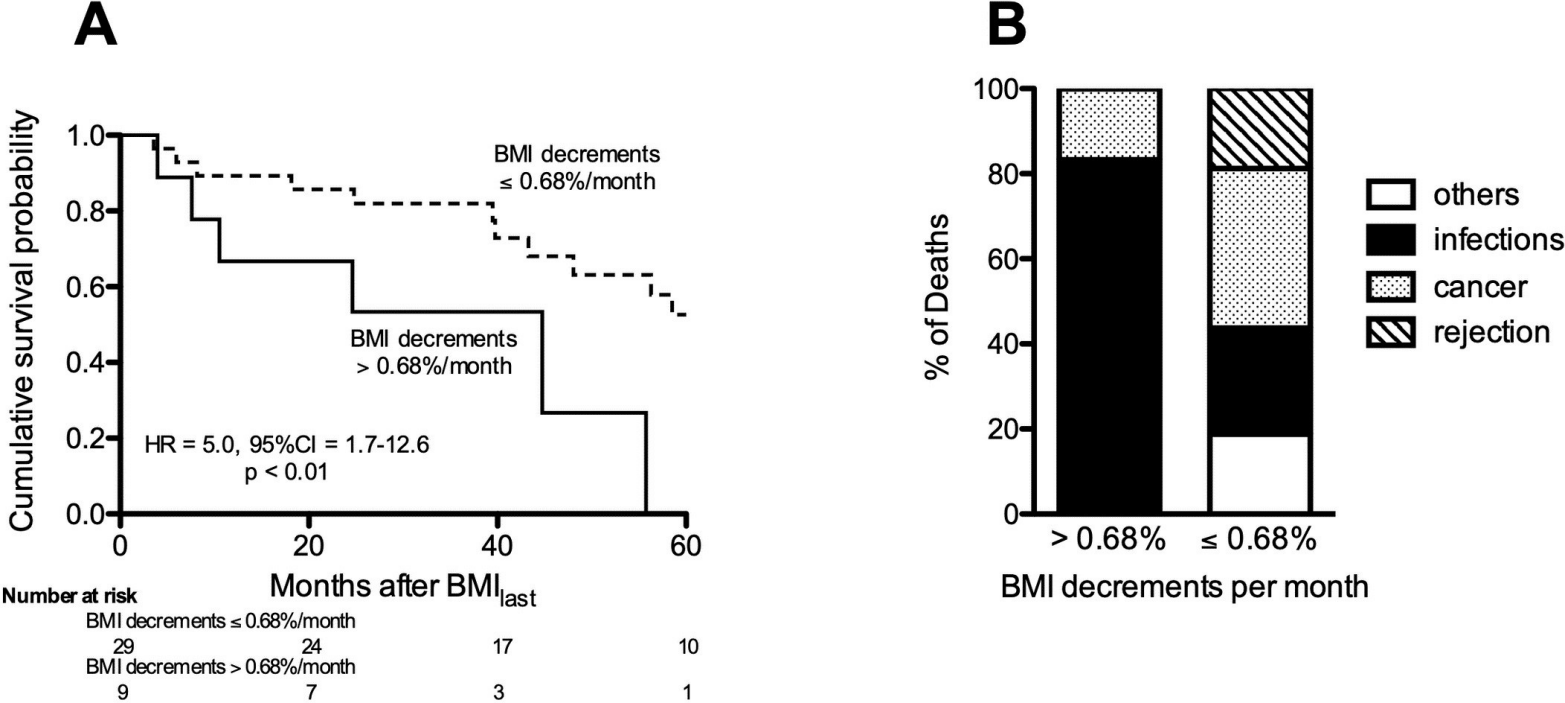
Post-transplant mortality. **A)** Mortality among IPF patients with pre-transplant BMI decrements >0.68%/month was increased after their transplantations. **B)** Causes of deaths among these transplant recipients tended to be associated with their earlier, pre-transplant BMI changes.

Stratification of the transplant recipients into those with pre-transplantation BMI decrements exceeding 0.68%/month *vs*. those with more stable BMI showed survival of the latter were significantly better (Fig 2A). Subjects with decrements of BMI_delta_ greater than 0.68%/month in the year preceding lung transplant had more than 4-fold increased risk of death compared to those with lesser BMI_delta_ decrements (HR = 4.6; 95%CI 1.7–12.6, p = 0.003). Deaths attributable to infections predominated in those with the greatest pre-transplant BMI_delta_, whereas mortality due to cancers or chronic allograft failure was increased among those with more stable BMI (Fig 2B).

### Circulating adiopokine levels

**Fig 3:**
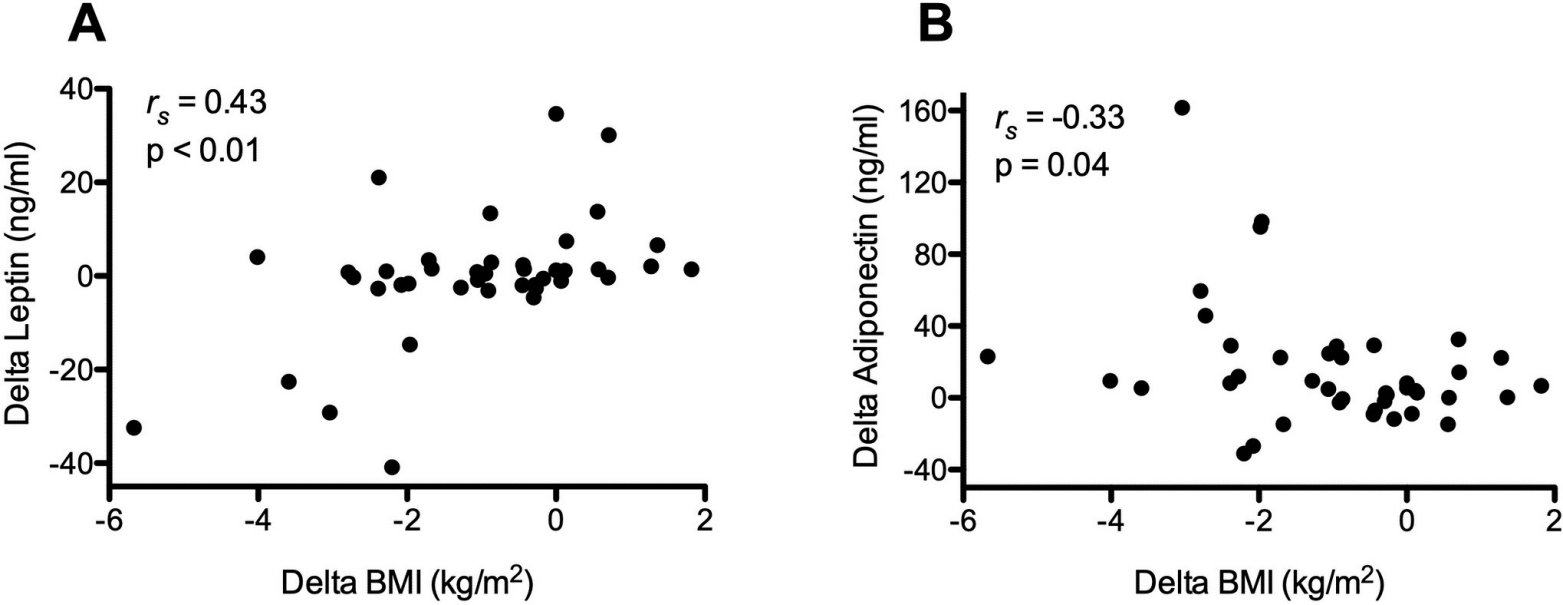
Correlations of body mass index and adiponectins. **A)** Changes in leptin concentrations were correlated with corresponding changes of BMI during the intervals from BMI_first_ to BMI_last_. **B)** Changes in adioponectin concentrations were inversely correlated with corresponding changes of BMI during the intervals from BMI_first_ to BMI_last_.

Plasma samples were available from 92 UPMC patients that had been obtained at the same time as BMI_first_ (n = 69), or BMI_last_ (n = 64), or both BMI determinations (n = 41). Leptin concentrations correlated with corresponding BMI_first_ (*r*_*s*_ = 0.7, p<0.01) and BMI_last_ (*r*_*s*_ = 0.5, p<0.01). Longitudinal BMI changes also correlated with concomitant alterations of plasma leptin (Fig 3A). There were no associations of longitudinal change in leptin levels with TFS (HR = 1.0; 95%CI 1.0–1.02, p = 0.3).

Cross-sectional adiponectin levels did not correlate with either BMI_first_ (*r*_*s*_ = -0.12, p = 0.31) or BMI_last_ (*r*_*s*_ = -0.12, p = 0.34). Longitudinal changes of adiponectin levels were modestly correlated [inversely] with corresponding BMI_delta_ (Fig 3B). Adiponectin levels were also unrelated to concomitant leptin values at BMI_first_ (*r*_*s*_ = 0.05, p = 0.69) and BMI_last_ (*r*_*s*_ = -0.03, p = 0.81). There were no significant associations of longitudinal changes in adiponectin levels with TFS (HR = 1.0; 95%CI 1.0–1.02, p = 0.2).

### BMI and CD4 T-cell Differentiation

**Fig 4:**
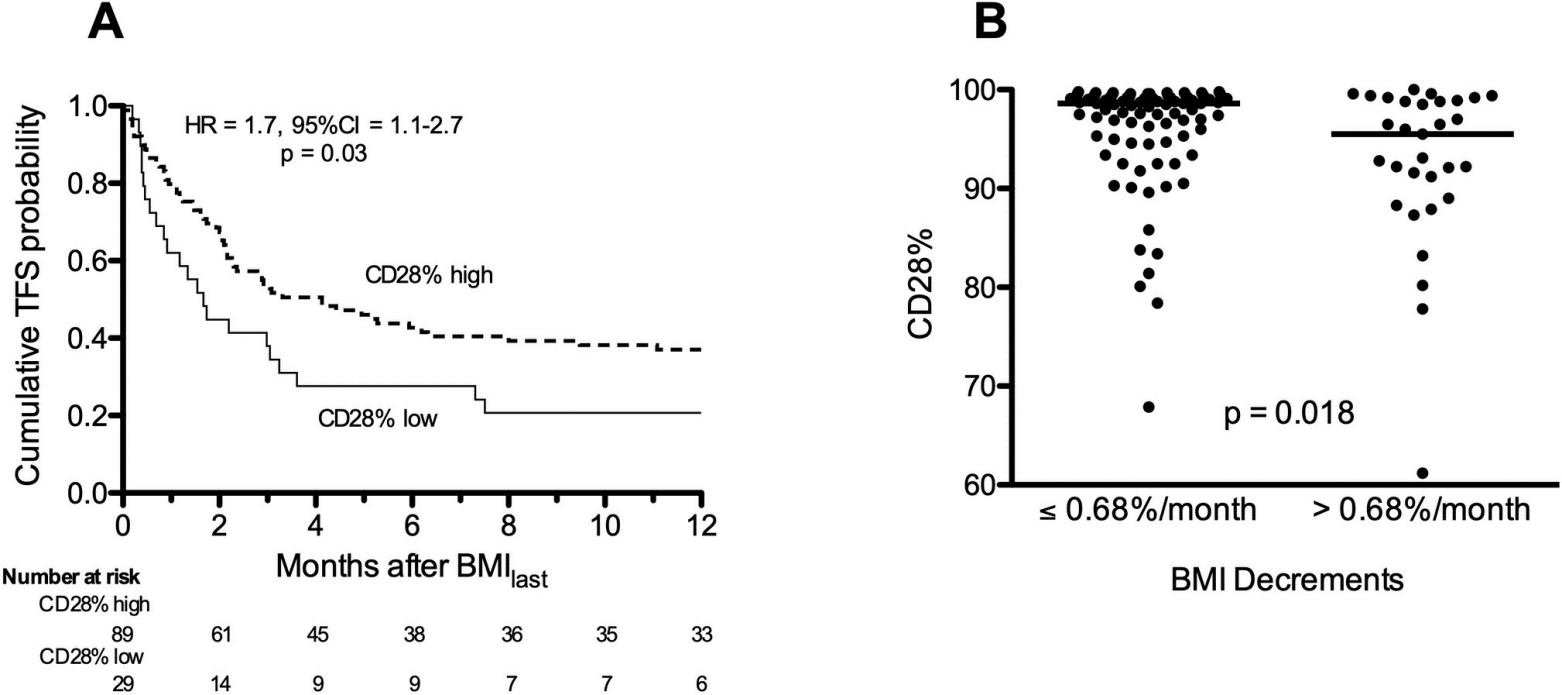
Correlation of body mass index and CD4 T-cell differentiation. **A)** Transplant-free survival (TFS) was worse among IPF subjects with low CD28 expression compared to subjects with high CD28%. **B)** The proportion of CD4 T-cells that express CD28 (CD28%) at BMI_first_ was diminished among the quartile of IPF subjects with greatest BMI decrements.

In order to explore possible linkages between weight loss and immune activation, as described in other populations [20], we analyzed CD4 T-cell CD28 expression relative to BMI determinations. CD4 T-cells were examined at BMI_first_ in 120 discovery cohort subjects. Survival analyses confirmed low [abnormal] CD28% was associated with decreased one-year TFS compared to subjects with normal CD28% (Fig 4A) (23), which persisted after multivariable adjustment (HR = 1.8, 95%CI = 1.1–3.1, p = 0.04).

CD28% were significantly reduced (more abnormal) in the IPF subjects who subsequently had the greatest BMI decrements (Fig 4B).

## Discussion

These data show that BMI decrements among IPF patients are associated with increased risks of transplantation and/or death. This relationship was evident in analyses of BMI changes as percentages of initial values, and with BMI changes as functions of time. Moreover, these findings were very similar in two distinct cohorts (Fig 1A and 1B) and independent of pulmonary physiological parameters that are widely used in IPF prognostic indices [2, 3], or age, or initial BMI. This study further demonstrated a potential link for adipokine dysregulation and immune activation in association with BMI decrements and poor outcomes in IPF patients. Most importantly, the present data indicate monitoring BMI may have prognostic value in IPF patients (Fig 1A and 1B). The robustness and reproducibility of the findings here in relatively large, independent study populations defined by contemporary criteria illustrate the importance of longitudinal BMI reductions as a mortality risk in IPF, even in patients who are variously under- or over-weight at their initial diagnosis.

Weight loss is a harbinger of poor outcomes in several chronic conditions including COPD, rheumatoid arthritis, congestive heart failure, coronary artery disease, asthma and bronchiectasis [12–14, 26, 27] and has recently been reported in association with mortality in IPF [15–18]. Baseline BMI was reported to be associated with survival in a single center IPF cohort [15]. In contrast to that earlier report, more recent studies link longitudinal weight loss, but not baseline BMI, to survival in IPF patients [17, 18]; analogous to our findings (Fig 1A and 1B).

In distinction to these reports, however, we explored additional characteristics of BMI decrements in IPF. A novel observation of the present study is the effect of pre-transplant BMI decrements on IPF mortality after lung transplantations (Fig 2A). This finding is contrary to prior suggestions of better survival in association with pre-transplant weight loss in patients with various chronic lung diseases [16]. However, patients in those earlier reports intentionally lost weight by voluntary dietary regulation and exercise, which is contrary to the weight loss in our IPF population which was anecdotally observed in almost all cases to be unintended. Although subject numbers are small here, the present data indicating trends between pre-transplant BMI changes and causes of later mortality (Fig 2B), also suggests weight loss may be related to (or possibly reflect) systemic processes that influence host defense.

Accumulations of CD28^null^ effector-memory T-cells are a specific consequence of repeated antigen receptor activation by conventional peptide epitopes and are not simply due to nonspecific global inflammation [23, 24, 28]. IPF subjects with the greatest BMI decrements, and worst prognoses, had the most marked CD4 T-cell end-differentiation (Fig 4B). Although the role of immune processes in IPF has been controversial, several recent studies have shown adaptive immune activation among many of the patients afflicted with this lung disease, and in several examples these immunologic abnormalities are highly related to clinical manifestations and patient outcomes [23, 29–35]. While cause and effect cannot be established from the present findings, these data are also consistent with findings in other populations that link adaptive immune activation and cachexia [20]^.^

Adipokines such as leptin and adiponectin are proteins secreted predominantly by adipocytes that can modulate body weight via their effects on metabolic regulation [36]. Adiponectin expression has been recently shown to be reduced in lungs of IPF patients [37]. Changes in circulating adipokine concentrations are adversely linked to lung function abnormalities in COPD populations [38, 39] and also associated with various effects on the adaptive immune system [40]. While changes in leptin and adiponectin levels were correlated with BMI decrements here (Fig 3A and 3B), we did not identify significant relationships of these adipokines with survival. The regulation of these cytokines is complex, however, and is variously affected by poorly understood mechanisms, particularly in conditions of disease. As examples, leptin production is increased by acute phase reactants and other immune mediators [41], and circulating concentrations of this adipokine are typically higher in conditions characterized by acute or chronic inflammation [42]. Adiponectin levels are generally decreased in the context of the low-level chronic inflammation that is associated with obesity, but may be unchanged or even increased in other inflammatory conditions [43]. The lack of an overt relationship between adipokines and disease progression has also been described in several other syndromes [44].

The present report describes our initial investigation into the association of BMI changes with outcomes among IPF patients with IPF. It is not a definitive validation of the BMI_delta_ for clinical use, and to attempt a rigorous comparison with other prognostic indices was beyond the intent and scope of this study. Instead, this analysis was prompted by clinical observations that led us to hypothesize BMI_delta_ reductions may be a significant, if under-appreciated, portent of poor outcomes in IPF patients. Moreover, after having found our hypothesis was plausible, incremental studies were performed to explore underlying mechanisms that might account for the BMI_delta_ and, more importantly, possibly contribute to disease progression.

This report has limitations. By necessity it is a retrospective analysis, and has the inherent flaws of these study types. Cryptic biases are possible, such as over-representations of more healthy survivors, due to premature subject drop out of the most ill patients prior to their second BMI measures. Although the aggregate subject number here is greater than in analogous reports [17, 18], the power of future investigations will be enhanced by even larger group sizes and more frequent collection of prospective, longitudinal data and biological specimens. Adipokine analyses of small populations seem particularly liable to confounding due to the complexities of their regulation. Details about dietary modifications (intended to gain or lose weight) will enhance further studies. The subjects here were analyzed prior to the introduction of anti-fibrotic medications [45], and it is possible those agents could have various effects on dietary intake, weight loss, and/or survival that will need consideration in future investigations. Causes of death in the non-transplanted IPF patients here were often not available, and more stringent mortality assessments in larger subject numbers could illuminate clinically-significant relationships, as seen in the lung transplant recipients (Fig 2B). Despite these limitations, however, the present findings may prompt considerations to test the incorporation of BMI_delta_, perhaps in addition to other demographic and physiological parameters [2, 3, 5, 6, 8–10, 25], in the future development and validation of indices that could enrich and enhance our ability to predict the course of IPF in individual patients.

## Conclusions

IPF patients with more stable BMI had longer transplant-free survival than patients with greater weight decrements in both IPF study cohorts. These findings could be an impetus for further research to more fully determine causes of the weight loss in IPF patients and better understand the pathological mechanism(s) that link BMI_delta_ to progression and outcome of this lung disease. The present data also indicate changes of BMI *per se*, or in combinations with other measures, could eventually be a useful clinical tool to identify IPF patients with increased risks of near-term mortality.

## Supporting information

S1 Data Data utilized for the analysis of this study. (XLSX)
